# Enhanced Visualisation of Normal Anatomy with Potential Use of Augmented Reality Superimposed on Three-Dimensional Printed Models

**DOI:** 10.3390/mi13101701

**Published:** 2022-10-10

**Authors:** Jade Geerlings-Batt, Carley Tillett, Ashu Gupta, Zhonghua Sun

**Affiliations:** 1 Discipline of Medical Radiation Science, Curtin Medical School, Curtin University, Perth, WA 6845, Australia; 2 Curtin HIVE (Hub for Immersive Visualisation and eResearch), Curtin University, Perth, WA 6845, Australia; 3 Department of Medical Imaging, Fiona Stanley Hospital, Perth, WA 6150, Australia; 4 Curtin Health Innovation Research Institute (CHIRI), Curtin University, Perth, WA 6845, Australia

**Keywords:** anatomy, anatomy education, three-dimensional printing, augmented reality, Microsoft HoloLens 2, enhanced visualisation, simulation

## Abstract

Anatomical knowledge underpins the practice of many healthcare professions. While cadaveric specimens are generally used to demonstrate realistic anatomy, high cost, ethical considerations and limited accessibility can often impede their suitability for use as teaching tools. This study aimed to develop an alternative to traditional teaching methods; a novel teaching tool using augmented reality (AR) and three-dimensional (3D) printed models to accurately demonstrate normal ankle and foot anatomy. An open-source software (3D Slicer) was used to segment a high-resolution magnetic resonance imaging (MRI) dataset of a healthy volunteer ankle and produce virtual bone and musculature objects. Bone and musculature were segmented using seed-planting and interpolation functions, respectively. Virtual models were imported into Unity 3D, which was used to develop user interface and achieve interactability prior to export to the Microsoft HoloLens 2. Three life-size models of bony anatomy were printed in yellow polylactic acid and thermoplastic polyurethane, with another model printed in white Visijet SL Flex with a supporting base attached to its plantar aspect. Interactive user interface with functional toggle switches was developed. Object recognition did not function as intended, with adequate tracking and AR superimposition not achieved. The models accurately demonstrate bony foot and ankle anatomy in relation to the associated musculature. Although segmentation outcomes were sufficient, the process was highly time consuming, with effective object recognition tools relatively inaccessible. This may limit the reproducibility of augmented reality learning tools on a larger scale. Research is required to determine the extent to which this tool accurately demonstrates anatomy and ascertain whether use of this tool improves learning outcomes and is effective for teaching anatomy.

## 1. Introduction

Understanding anatomy is essential for many healthcare disciplines. Learning anatomy can be time consuming and costly, and usually begins with exposure to videos, diagrams, and plastic models [1]. However, these tools are relatively non-interactive, with their value generally further restricted by their lack of realism. While more advanced anatomy and physiology has traditionally been taught using cadaveric (wet) specimens and plastinated models, virtual reality (VR) and augmented reality (AR) are becoming more prevalent teaching tools and have the potential to improve student outcomes [2,3,4,5]. The Anatomage was the first virtual dissection table and has existed since 2004 [6]. Anatomage tables allow users to interactively control and dissect a life-sized, virtual, realistic visualisation of a human cadaver [1,6], with its likeness to wet specimens making it a viable substitute for cadavers in dissection courses and radiology education [1].

Mixed reality (MR) is characterised by the supplementation of real-world objects with virtual objects through the use of AR [7]. Moreta-Martinez et al. employed similar methods to those utilised in this project, using 3D Slicer to segment a computed tomography (CT) dataset, as well as Unity 3D for AR development and app deployment [8]. However, this MR tool required a smart device which users generally dedicated at least one hand to holding, limiting the user’s experience. Alternatively, Maniam et al. deployed their application to the Microsoft HoloLens, allowing users to manipulate 3D printed temporal bone models with both hands [7]. The Magic Mirror is another AR tool available for teaching anatomy, using a depth camera similar to the Microsoft Kinect to track users standing in front of a screen display; it augments a CT dataset onto the user, creating the illusion of the user looking inside their body [1,9,10]. The Magic Mirror is also capable of presenting text information, as well as additional images of anatomy, in order to inform the user’s understanding of spatial relationships [1,9,11]. 

AR and MR tools may also be useful for physicians by improving education and training, and through creating patient-specific, customisable tools to provide guidance during interventional procedures [2,3,4,5,8]. Although the demand and application of MR technology in clinical environments is increasing, it is rarely seen in educational settings [7]. The current literature indicates that segmentation from magnetic resonance imaging (MRI) datasets is uncommon, and realistic 3D printed models instead tend to be derived from CT datasets [1,4,9,12]. The technology is available to create VR, AR, and MR teaching tools on a larger scale. Despite this, their widespread application is relatively restricted, largely due to the high cost and limited accessibility associated with the necessary software and hardware, as well as the interdisciplinarity required to develop these tools. However, introducing VR, AR, and MR as complimentary tools for teaching anatomy may stimulate more immersive, interactive, student-centred learning environments [11,13,14], ultimately improving student outcomes and creating more knowledgeable health professionals [11,14]. This was the motivation for conducting this study, with the aim of creating a tangible learning tool that can be viewed, held, and manipulated by students or healthcare professionals learning anatomy. The purpose of this study was to facilitate a more interactive, immersive learning experiences by using these innovative 3D visualisation tools. This study aimed to utilise an MRI dataset to take advantage of the improved visualisation of soft tissue compared to CT, with the potential to derive more realistic physical and virtual simulated anatomical models of these structures in addition to bony anatomy. 

## 2. Materials and Methods

**Figure 1:**
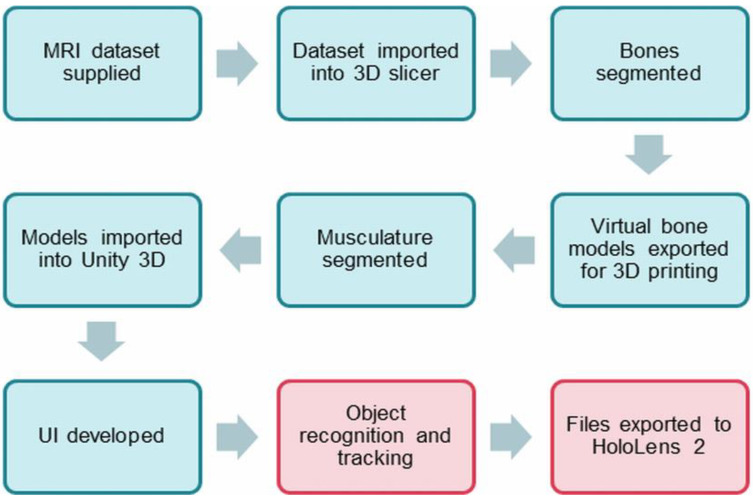
Process diagram involved in the study. Red sections refer to the steps that are ongoing for further development.

Figure 1 is a flow chart showing the steps that were undertaken in this study.

### 2.1. Image Processing and Segmentation of MRI Dataset Using 3D Slicer

An anonymised high-resolution left ankle T1 MRI dataset was acquired from a healthy volunteer with normal anatomy. Analyze 14.0 (AnalyzeDirect, Inc., Lexana, KS, USA) was originally used to segment data on a laptop operating Windows 7 with an integrated graphics card. Analyze 14.0 is a program often used by academics involved in biomedical research and is capable of performing image post-processing, visualisation, and segmentation [15,16,17]. However, segmentation with Analyze 14.0 was difficult and unnecessarily time consuming due to relying to a great extent on manual input for image segmentation. Instead, 3D Slicer, an open-source software with numerous tutorials available online and a simple user interface (UI), was used to segment the dataset. The dataset was inadvertently horizontally inverted during import into 3D Slicer, with the subsequent virtual and 3D printed models essentially of right feet and ankles. 

### 2.2. Segmentation of Bony Anatomy

**Figure 2:**
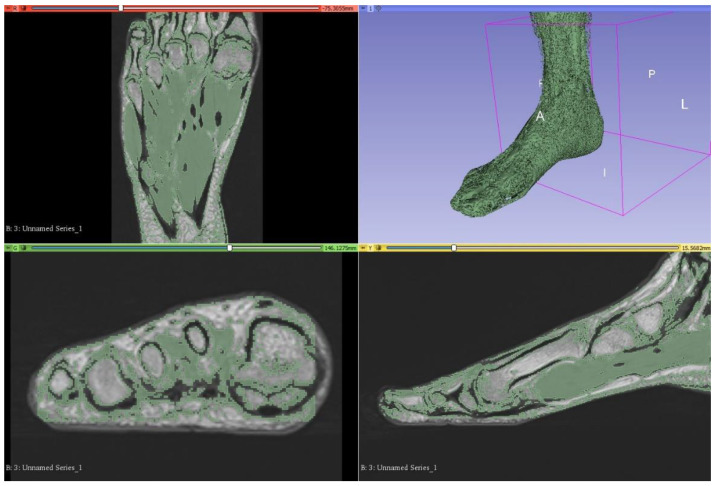
Attempted segmentation of musculature in 3D Slicer via the thresholding function, which proved ineffective. This is as viewed on the 3D Slicer observation panel, which is organised into axial (**upper left**), 3D (**upper right**), coronal (**lower left**), and sagittal views (**lower right**). A—anterior, I—inferior, L—left, P—posterior, R—right.

Unlike CT datasets, signal intensities (grey level) on MRI can be similar between different structures such as bone and musculature. Since bone appears white on CT, semiautomatic segmentation according to attenuation (grey level) thresholding is an effective method for bony segmentation of CT datasets. However, bony detail on MRI is often analogous with several other tissue types, such as subcutaneous and intermuscular adipose tissue, and thresholding was consequently not a viable option for segmentation of this foot and ankle dataset (Figure 2). Although numerous open-source plugins were available for streamlining segmentation using 3D Slicer, there were none available for segmenting musculoskeletal anatomy from MRI datasets, specifically. Those designed for segmenting MRI datasets are generally related to brain imaging and were unapplicable in this case. Hence, manual methods were heavily relied upon to achieve accurate segmentation. 

**Figure 3:**
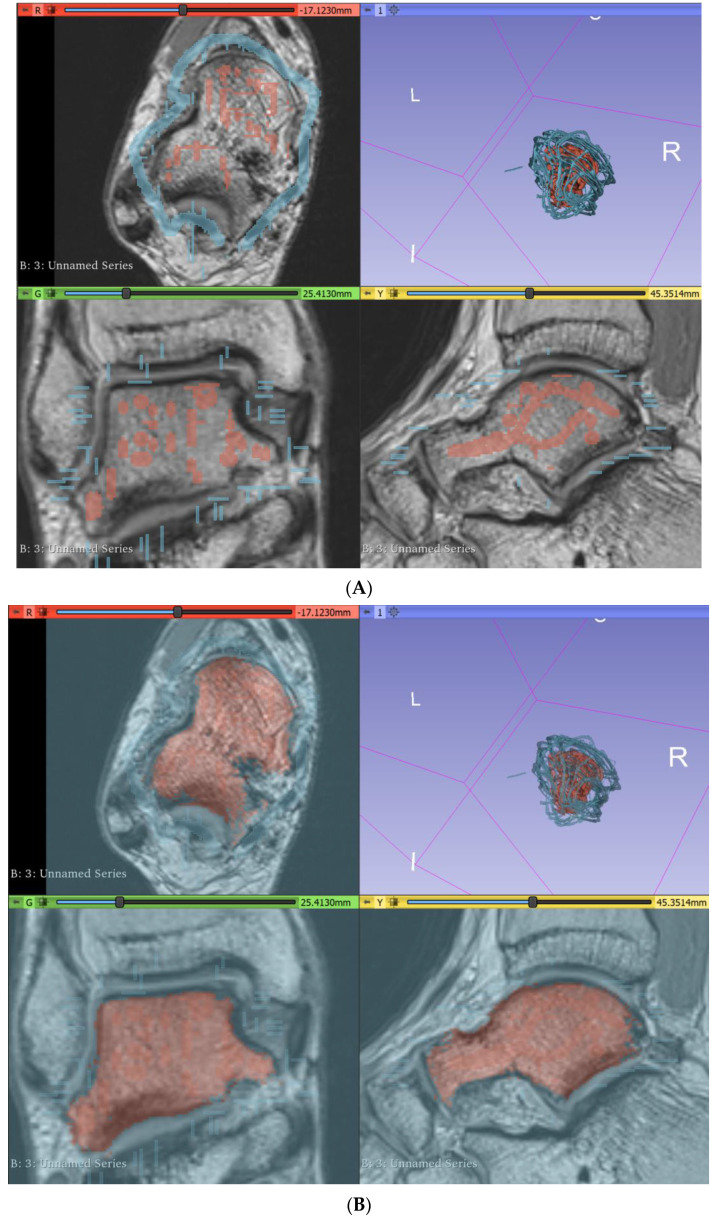
Segmentation of the talus via the “grow with seeds” function in 3D Slicer. (**A**) Seeds planted in the talus (red) and around the talus (blue). (**B**) Resultant segmentation layers. I—inferior, L—left, R—right.

**Figure 4:**
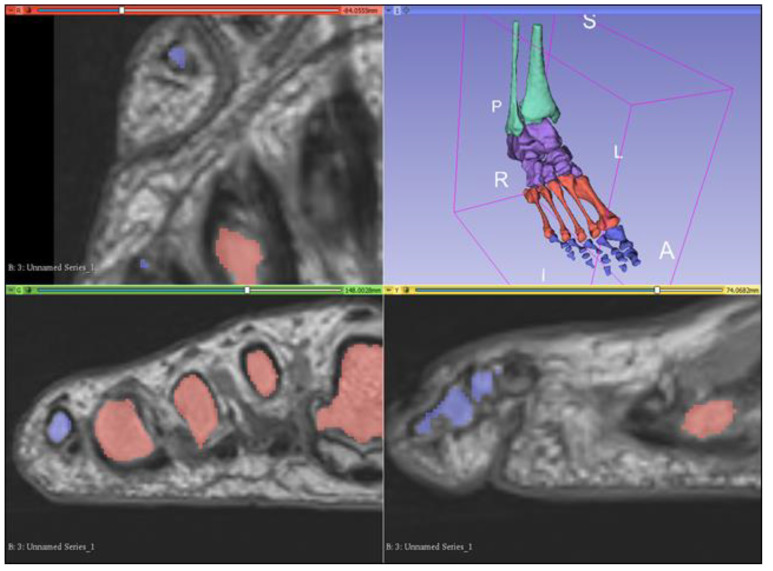
Completed bony model as viewed on the 3D Slicer observation panel. Bones are grouped into leg bones (green), tarsals (purple), metatarsals (red), and phalanges (blue). A—anterior, I—inferior, L—left, P—posterior, R—right, S—superior.

The “grow with seeds” function was predominantly used to achieve segmentation of bony structures. This involved crudely placing seeds within the structure of interest on one segmentation layer, as well as placing seeds on a different segmentation layer around the structure of interest to distinguish its boundaries (Figure 3). This allows 3D Slicer to more accurately segment according to the image threshold. This process worked relatively well and was repeated to segment each bone. Fine adjustments were then made using the paint, erase, and median smoothing tools to correct defects and extrusions on the models’ surfaces. Although universal smoothing provided a fast alternative to manual smoothing brushes, these functions also removed relevant bony prominences from the virtual models, with ridges and grooves corresponding to muscle attachment points, such as those on the sustentaculum tali, inadvertently removed. Hence, smoothing kernels could not be applied universally to the models. The resultant models also had a “lumpy” appearance, with Gaussian smoothing brushes used on the 3D virtual bones to manually remove lumps appearing to be artefacts of the segmentation process. The completed bony model (Figure 4) was exported as .STL (Standard Tessellation Language) files and sent for 3D printing. Bones on the virtual models were connected prior to 3D printing. One miniature prototype was printed in an acrylic resin and another life-size model was printed in white Visijet SL Flex, with a supporting base attached to the plantar aspect of the model (24 × 9 × 22 cm). 

### 2.3. Segmentation of Musculature

**Figure 5:**
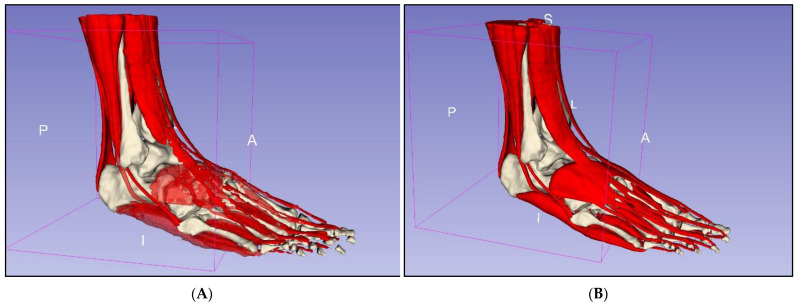
Segmentation of musculature. (**A**) Rough segmentation. (**B**) Segmented musculature after manual refinement. A—anterior, I—inferior, L—lateral, P—posterior, S—superior.

**Figure 6:**
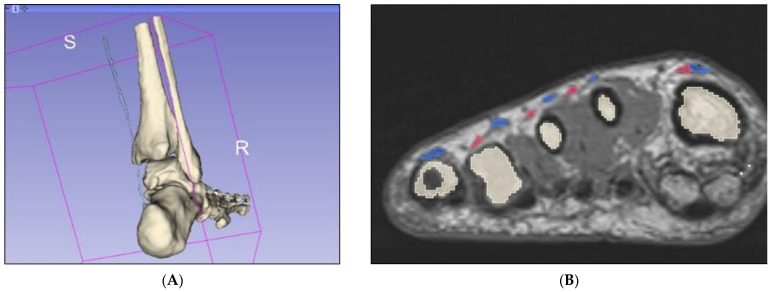
Segmentation of thin tendons. (**A**) Plantaris tendon segmented via interpolation method only. (**B**) Coronal slice of midfoot demonstrating long (pink) and short (blue) extensor tendons of the digits. R—right, S—superior.

Segmenting musculature presented similar challenges to segmenting bony anatomy, and thresholding alone resulted in frequent inclusion of irrelevant structures. However, thresholding was useful for masking and allowed for more precise painting. Masks were created for low signal intensities (black) and the paint brush was used to paint over a tendon every few axial slices. The “fill between layers” function was then used to interpolate between slices and create 3D models for the tendons. On a new segmentation layer, the threshold was adjusted for the moderate signal intensity (grey) corresponding to muscles, with the same method used to roughly segment muscles. Tendon and muscle segmentation layers were then merged, with the global “closing” smoothing tool used to fill defects within the model. This process was repeated for each muscle and associated tendons, with the paint, erase, and smoothing brushes subsequently used for fine adjustment of the models’ surfaces (Figure 5). The interpolation function did not work well for segmenting thin tendons such as the plantaris tendon, as well as the flexor and extensor tendons of the digits, which were only two voxels in width at points (Figure 6). Hence, manual paint and erase tools were primarily used to segment these smaller structures.

### 2.4. Creation of Mixed Reality

**Figure 7:**
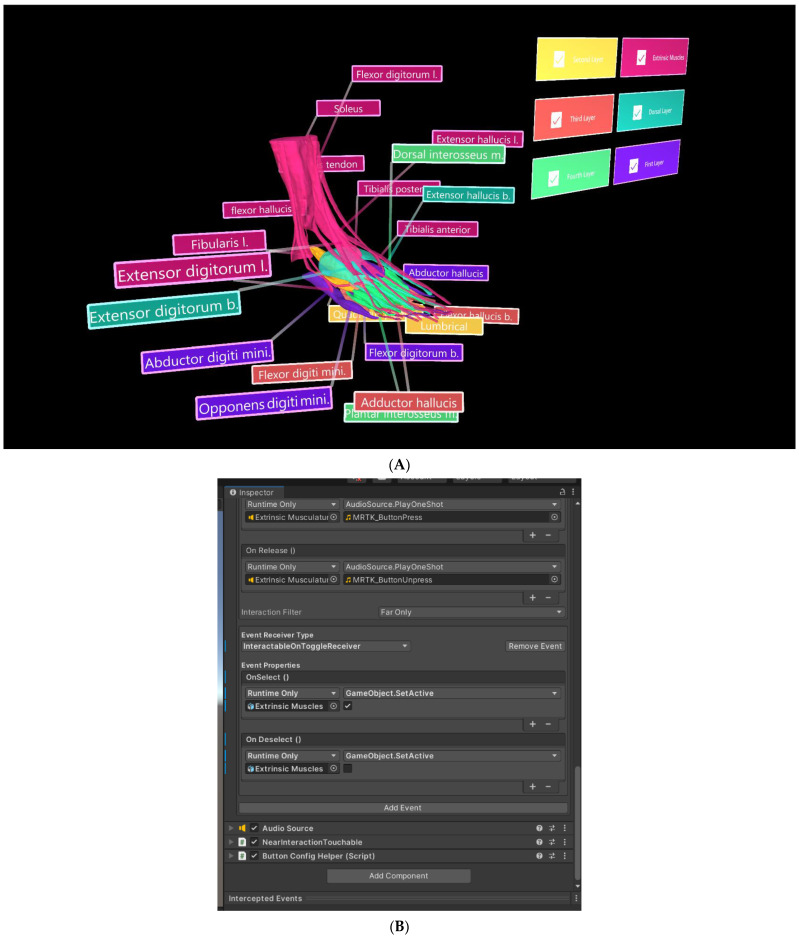
Muscle objects imported into Unity 3D, where UI was also developed. (**A**) Toggle switch used to selectively display the “Extrinsic Muscles” object. (**B**) Inspector panel for the toggle switch associated with the “Extrinsic Muscles” object.

The segmented muscle objects were exported in groups—extrinsic muscles, dorsal layer, and first, second, third, and fourth plantar layers—in object file (.OBJ) format and imported into Unity 3D (V2020.3.26[LTS], San Francisco, CA, USA). Each muscle group was assigned a different colour to improve distinguishability, with six “PressableButtonHoloLens2ToggleCheckBox_32 × 96” from the “Mixed Reality Tool Kit Foundation Package” [18] used as toggle switches for selectively displaying each muscle group (Figure 7). In the inspector panel, the actions of engaging and disengaging the toggle switches were assigned the default sounds “MRTK (Mixed Reality Took Kit)_ButtonPress” (Microsoft, version: 1.0.2209.0, Redmond, DC, USA) and “MRTK_ButtonUnpress” [18], respectively. To display and hide muscle layers, event properties were adjusted for each toggle switch, as displayed in Figure 7A. Tooltips from the MRTKFoundation Package were also used as labels to identify each muscle. 

A Vuforia HoloLens 2 (Vuforia Engine 10.10.2, Boston, MA, USA) sample scene [19] was used as a template for the AR component of this project, with musculature objects, toggle switches, and labels imported into the scene as assets. The Vuforia Object Scanner, a mobile phone app, was used for object recognition. Using the mobile phone’s camera, the app mapped the object and created an object target for import into Unity 3D. Microsoft’s Visual studio was used to export the Unity 3D file as an application able to be read and executed by the HoloLens 2. 

## 3. Results

**Figure 8:**
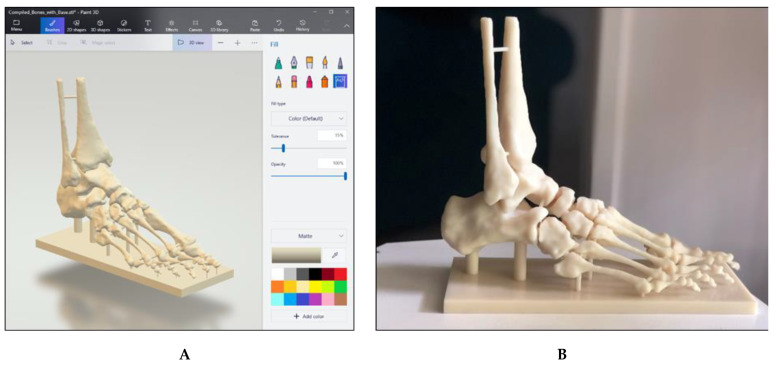
Virtual and 3D printed bony models. (**A**) Bony model in .STL file with painted connections and supporting base. (**B**) Life-size model printed in white Visijet SL Flex (Polyjet 6000 HD, 3D Systems, Melbourne, Australia) with supporting base.

Virtual models (.OBJ and .STL filetypes) were created as a result of successful segmentation of the high-resolution MRI dataset, representing musculature and bony anatomy of the ankle and foot. These models can be viewed as .OBJ or .STL filetypes via software such as Microsoft Paint 3D (Figure 8A), while .STL files can also be used for 3D printing. Two additional virtual bony models were created prior to 3D printing, with one consisting of bones connected by small bridges and another including a supporting base attached to the plantar aspect of the foot (Figure 8B).

**Figure 9:**
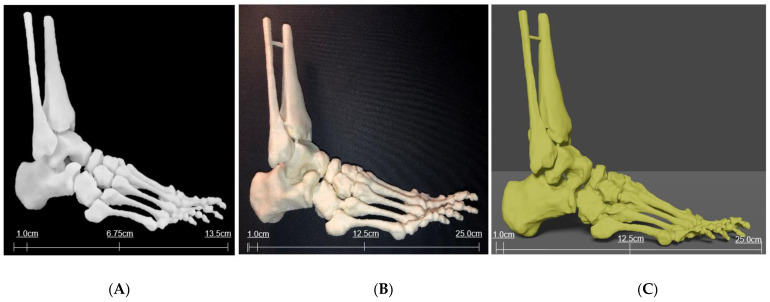
Three-dimensional printed models of foot and ankle bony anatomy in white acrylic resin (**A**) using Formlabs 3D printer (Massachusetts, USA) and TPU (**B**) and PLA (**C**) using Ultimaker 2 Extended 3D printer (Ultimaker BV, Geldermalsen, the Netherlands).

One miniature prototype and three life-size models were 3D printed in various materials. The prototype was printed in a white acrylic resin (Figure 9A), while one life-size model (25 × 9 × 19 cm) was printed in flexible white thermoplastic polyurethane (TPU) (Figure 9B) and one was printed in yellow polylactic acid (PLA) (Figure 9C). Another life-size model (24 × 9 × 22 cm) was printed in white Visijet SL Flex, with a supporting base attached to the plantar aspect of the model (Figure 8B). 

**Figure 10:**
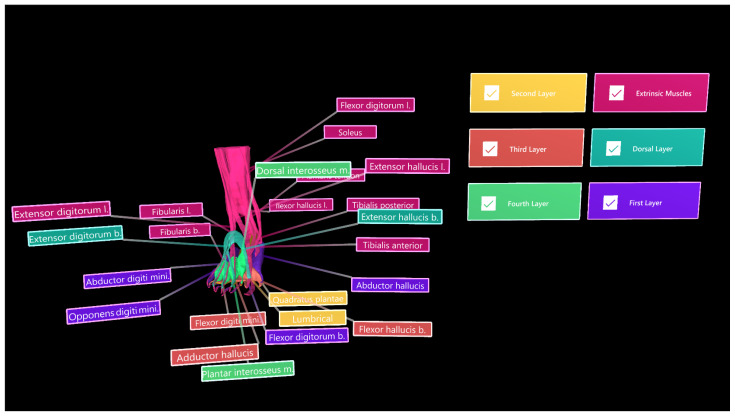
Muscle objects imported into Unity 3D for demonstration of different groups/layers. Toggle switches and colour-coded extrinsic muscle group (magenta), dorsal layer (light blue), and first, second, third, and fourth plantar muscle layers.

Muscle objects were successfully imported into the Vuforia HoloLens 2 sample scene in Unity 3D (Figure 10), and toggle switches and labels functioned as expected. However, the Vuforia object tracker did not function as intended and object recognition and AR superimposition was not achieved. A video was generated to show segmentation and interaction of bones and muscles in Unity for potential demonstration of AR views (Supplementary File S1).

## 4. Discussion

This study showed that accurate segmentation of bony foot and ankle structures was achieved, as reinforced by an experienced radiologist, who was able to recognise several bony prominences. However, the exact degree of accuracy cannot be determined by this study alone as our focus was to develop a workflow of image processing and segmentation of bony and muscular structures for 3D printing and AR visualisation. Future research could quantify and investigate the accuracy of this method of segmentation by using imaging techniques such as CT to compare the radiographic appearance of real bony anatomy with printed anatomy. Alternatively, a tool such as that developed by Taha and Hanbury [20] could be used to evaluate the accuracy of segmentation. All but one of the relevant muscles were visualised in the MRI dataset. Fibularis (peroneus) tertius is situated superficially in the anterior compartment of the leg and is involved in ankle eversion and foot dorsiflexion [21]. However, cadaveric studies have determined fibularis tertius to be absent in some individuals, with the prevalence of the muscle varying between populations [22]. Fibularis tertius was not visualised in the supplied MRI dataset, which may be due to the muscle being absent in this particular individual, and it was consequently unable to be segmented.

Despite segmenting a high-resolution MRI dataset, some tendons were only a few voxels in width, and semiautomatic segmentation tools could not be solely relied upon to achieve satisfactory results. Additionally, most semiautomatic segmentation tools were designed for CT datasets and used some form of thresholding; hence, semiautomatic segmentation was not appropriate for segmentation of this dataset. This was also true for segmentation plugins, which were largely designed for CT datasets or non-musculoskeletal MRI datasets and were not applicable for use in this study. For example, several plugins intended to assist in segmentation of the brain from MRI datasets were available, but did not yield desired outcomes when applied to the foot and ankle dataset. However, the use of an MRI dataset was necessary for proper visualisation of soft tissue, musculature, and tendons, in addition to bony structures, which would otherwise not have been possible using a CT dataset. Hence, a heavy reliance on manual segmentation operators was applied for this project. Future studies should endeavour to properly and quantitatively assess the accuracy of available semiautomatic segmentation tools, comparing their usefulness for MRI versus CT datasets.

The virtual models appeared to resemble the original MRI dataset and adhered to the illustrations and diagrams provided in multiple anatomy textbooks [21,23]. For example, the tendons of extensor digitorum longus and brevis were at times difficult to distinguish from one another. However, the final segmentations matched the models provided by Kelly and Peterson, with short extensor tendons situated medially to the long extensor tendons [23]. Although the virtual models appeared anatomically correct, reliance on manual segmentation tools required personal interpretation, which has consequently predisposed their creation to bias and limited the reproducibility of this method. Additionally, some structures overlapped, while models for separating structures often occupied common voxels. Rectifying this problem required further personal interpretation, and the virtual models were resculpted via use of the manual paint and erase tools. Omission of this bias is possible through the use of technological tools. Algorithms and plugins should be developed to facilitate more automated segmentation of musculoskeletal MRI datasets; however, research would also be required to attain the accuracy of these tools.

Very few exploratory studies have used the HoloLens in a MR capacity; instead, they have simply used conventional AR. Maniam et al. successfully developed a MR tool incorporating the HoloLens and 3D printed models of temporal bones using Unity; however, they do not fully disclose their method for achieving object recognition and tracking [7]. Several review and scoping review articles reported the use of AR in anatomical education, but augmented objects included images and diagrams from books, as well as websites and cadavers, and varied between studies [24,25,26]. Our study used 3D printed personalised models derived from MRI data as augmented objects to develop a more advanced learning environment, but we only presented early findings on 3D printed models and image segmentation. Open-source tools for object recognition were difficult to locate for this project, with this technology generally inaccessible, which may be due to it being relatively new. Future studies are required to explore and evaluate the available tools for achieving high-quality object recognition and tracking, as well as assess the educational value of anatomy teaching and learning using our developed approach.

## 5. Conclusions

Bony foot and ankle anatomy and associated musculature was successfully segmented from a high-resolution MRI dataset. However, this was a time-consuming process due to the inaccuracy of semiautomatic segmentation processes and the consequent heavy reliance on manual paint and erase tools. Such extensive use of manual tools may have also introduced bias through personal interpretation and creation of the appearance of virtual models. Reproducibility of this method may have also been limited, since the outcome is likely dependent on the observer. This predisposition to potential bias could be overcome in future through the use of automated segmentation algorithms and plugins, such as those involved in machine learning or deep learning tools for rapid and efficient segmentation processes [27,28,29]. However, at present, there is a distinct lack of available plugins designed specifically for segmenting musculoskeletal MRI datasets. Further exploration and developments are required to create tools applicable to MRI datasets of the musculoskeletal system. 

Several virtual models and 3D printed bony models were created as a result of successful segmentation. Bony prominences were well-preserved on the 3D printed models and structures such as the sustentaculum tali were easily visualised. Although models were imported into Unity 3D and the UI was developed, this study was limited by an inability to achieve adequate object recognition and tracking for the HoloLens 2. The software required to develop these features is generally inaccessible in an open-source format, while the software identified for use in this study did not work as intended. The current literature exploring the development of mixed reality technologies appears to either not use the HoloLens or HoloLens 2, or not explicitly outline methods for achieving object recognition. Hence, future research should explore and evaluate the different tools available for creating these functions. With further technological improvements, this study could be advanced by persevering and working towards more reliable object recognition and tracking, in order to fully meet its primary objective. 

## Data Availability

The dataset used in this study is not publicly available due to strict requirements set out by authorized investigators.

## Supplementary Materials

Supplementary File S1: The following supporting information can be downloaded at: https://curtin-my.sharepoint.com/:v:/g/personal/282745h_curtin_edu_au/Ed8jMk7yC1ZBgjGhbipdlM4B7v_TAyeFzdoV-x2t1nSA8Q. This is a video link to demonstrate the segmented bones and muscles in Unity for interaction display and potential use for augmented reality views.
